# Endometrial Vascularization Characterized by Optical Coherence Tomography and Immunohistochemistry in Women Undergoing In Vitro Fertilization-Embryo Transfer Treatment

**DOI:** 10.3390/medicina55040081

**Published:** 2019-03-27

**Authors:** Tracy Sze Man Law, Wing Ching Cheung, Fangrong Wu, Ruizhe Zhang, Jacqueline Pui Wah Chung, Chi Chiu Wang, Xiaoyan Chen, Tin Chiu Li

**Affiliations:** 1 Assisted Reproductive Technology Unit, Department of Obstetrics and Gynaecology, The Chinese University of Hong Kong, Shatin, Hong Kong; tracylaw@cuhk.edu.hk (T.S.M.L.); cosycheung@cuhk.edu.hk (W.C.C.); wfr2015phd@link.cuhk.edu.hk (F.W.); zhangruizhe@link.cuhk.edu.hk (R.Z.); jacquelinechung@cuhk.edu.hk (J.P.W.C.); ccwang@cuhk.edu.hk (C.C.W.); tinchiu.li@gmail.com (T.C.L.); 2 Li Ka Shing Institute of Health Science, The Chinese University of Hong Kong, Shatin, Hong Kong; 3 School of Biomedical Science, The Chinese University of Hong Kong, Shatin, Hong Kong

**Keywords:** endometrium, optical coherence tomography, micro vessel, VEGF-A, implantation

## Abstract

*Background and objective:* Endometrial angiogenesis is a prerequisite for successful pregnancy. Optical coherence tomography (OCT) is a non-invasive physically optical imaging technique widely used in ophthalmology and cardiology. However, there is no study using OCT to evaluate endometrium. The aim of this study was to use OCT and traditionally histological methods to investigate endometrial vascularization in women undergoing in vitro fertilization-embryo transfer (IVF-ET) treatment and to determine the association with the pregnancy outcome. *Methods:* A total of 47 women were included in this study. OCT was used to assess endometrial vascularization by determining the high signal areas precisely on the seventh day after luteinizing hormone surge in non-conception natural cycles. Endometrial biopsies were obtained following OCT and immunohistochemistry was used to determine micro vessel and expression of vascular endothelial growth factor-A (VEGF-A) in the luminal epithelium, glandular epithelium and stroma, separately. Micro vessel counting was performed and the result was expressed as micro vessel density (MVD). A semi-quantitative H-score was used to determine the staining intensity of VEGF-A. *Results:* In women who successfully conceived after embryo transfer, the proportion of extensive high signal area in the uterine body detected by OCT (80%, 8/10), MVD (median number of micro vessels/mm^2^ of 10, range 4–17) and stromal expression of VEGF-A (median H-score of 189, range 72–395) were found to be significantly higher than those of women who did not conceive after embryo transfer in the subsequent IVF-ET treatment (OCT: 30%, 3/10; MVD: median number of micro vessels/mm^2^ of 7, range 4–10; VEGF-A: median H-score of 125, range 86–299, respectively). In addition, a significantly higher stromal expression of VEGF-A (median H-score of 196, range 84–395) and MVD (median number of micro vessels/mm^2^ of 9, range 5–16) was found in women with extensive high signal area in uterine body, compared to those with focal or no high signal area (stromal VEGF-A: median H-score of 135, range 92–302; MVD: number of micro vessels/mm^2^ of 6, range 4-11). *Conclusions:* Both immunohistochemistry and OCT demonstrated significant difference in vascularization of the peri-implantation endometrium between subjects who did and did not conceive after IVF-ET treatment. Our findings also suggest OCT appears to be a promising non-invasive or minimally invasive alternative to study endometrial vascularity in women with reproductive failure.

## 1. Introduction

A timely arrival of a viable embryo and receptive endometrium are two pre-requisites for successful pregnancy. In in vitro fertilization-embryo transfer (IVF-ET) treatment, implantation is considered to be successful when there is evidence of intrauterine gestational sac. There are several causes accounting for failed implantation, mainly including gamete/embryo factors and uterine factor. Angiogenesis is the formation of new blood vessels from existing vascular structures [1] and endometrium is the tissue where normal angiogenesis occurs, which plays an important role during the menstrual cycle, particularly around the time of embryo implantation [2]. Therefore, an alteration in endometrial vascularization may lead to unsuccessful implantation.

Optical coherence tomography (OCT) is a physically optical imaging technique widely used in ophthalmology [3] and cardiology [4]. There are few groups attempted to use OCT in female reproductive organs but they mainly focus on cervix, vagina and recently on ovary [5,6,7]. Tissue is illuminated by near-infrared light and the backscattered optical signal at micrometer-scale resolution, which approaches the resolution of histopathological examination. With recent development of OCT system, which incorporates catheter-based rotational probes, the fine probes could be inserted into the uterus to obtain non- or minimally-invasive, real-time, high-resolution, rotational positioning images. However, there is no data using OCT to evaluate endometrial characteristics, including endometrial vascularization, in vivo.

Traditionally histological measurement of micro vessel density (MVD) has been thought to be an accurate method to examine endometrial vascularization in several previous studies. One study has examined MVD in women with intrauterine adhesion and found that endometrial MVD in women recovering from transcervical resection of adhesions treatment was significantly higher than those non-responding to the treatment, which indicates that adequate angiogenesis is important for endometrial repair and function [8]. Another earlier study showed that endometrial MVD in the peri-implantation period of a non-conception cycle prior to embryo transfer in women with successful pregnancy was significantly higher than it in women with failed pregnancy, which suggested that MVD could be used as a tool to assess endometrial receptivity [9].

Vascular endothelial growth factor (VEGF)-A is one of the most important factors in angiogenesis regulation and it is synthesized in a phase-depending manner in human endometrium. It has been shown a three-fold increase of VEGF in the secretory phase, when compared to the proliferative phase [10], which suggested a crucial role of VEGF-A in the peri-implantation period. Our previous study has also showed an alteration in VEGF-A expression in different endometrial compartments from women undergoing ovarian stimulation and IVF-ET treatment [11].

In this study, our aim was to use OCT and immunohistochemistry to measure endometrial vascularization in the peri-implantation period in women undergoing IVF-ET treatment. We also investigated whether endometrial vascularization was associated with IVF pregnancy outcome.

## 2. Materials and Methods 

### 2.1. Ethical Issue

This study was approved by Joint Chinese University of Hong Kong - New Territories East Cluster Clinical Research Ethics Committee (CREC-2014.575 and CREC-2016.160). All the endometrial biopsies were collected with written informed consent of the participants. 

### 2.2. Subjects 

A total of 47 women undergoing IVF-ET treatment were included in this study. All of them had regular menstrual cycle and natural ovulation. Women who had uterine structural abnormalities or abnormal hystersalpingogram result were excluded in this study.

### 2.3. Optical Coherence Tomography 

In this study, although we have recruited 47 subjects, only 20 of them underwent examination by an optical coherence tomography imaging system (OCTIS) (Guangdong Winstar Medical Technology Co., China and Tomophase Inc., Cambridge, MA, USA)prior to the endometrial biopsy in this study, which was partly because of the unavailability of the OCTIS machine during a program update and machine maintenance and partly because of the unavailability of the person performing OCTIS. Nevertheless, the 20 women who underwent OCTIS represented a consecutive series of subjects, without bias in patient selection. OCTIS consists of an imaging computing system and a sterile detachable probe. The OCT probe is a catheter measures 2.5 mm in diameter and 15 cm in length which is sealed with a transparent outer sheath with a 1 mm long window near its tip for image scanning and capturing. Side-viewing probe was chosen as it is suitable for the imaging of tube-like hollow organs such as uterine cavity. There is a flexible fiber optical shaft for rotational imaging at 600–1200 rpm inside the sheath. The optical fiber is illuminated by a broad wavelength-range light source operating at 1300 nm and a sweeping rate of 50 kHz. The images acquisition speed is at 20 frames per seconds with 15 μm axial and 25 μm lateral resolution and depth up to 3 mm in both grey and color modes. The scanning window at the distal tip of the catheter is brought to the area of interest to capture OCT image. The OCTIS is United State Food and Drug Administration (K102599) approved for medical imaging. To obtain OCT images of endometrium, speculum examination was performed and OCT probe was inserted into the women’s uterus via cervix. The OCT probe was placed at the fundus of uterine cavity while simultaneously operating a pull-back along the uterine cavity to cervix to perform a scan of the whole uterine cavity. The OCTIS then created cross-sectional plane of endometrium which allowed real-time and off-time analysis of each section. Endometrial biopsy was performed afterwards. 

### 2.4. High Density Area Determined by OCT

**Figure 1:**
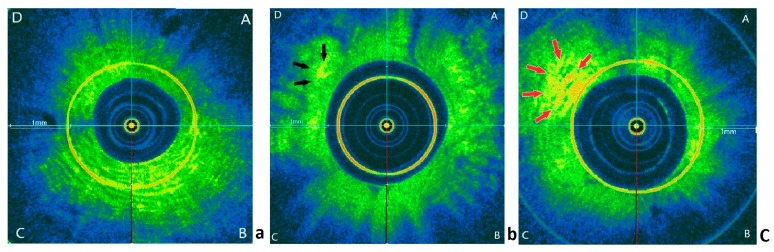
Representative OCT images for scoring of high signal area. (**a**) No high signal area. (**b**) Focal high signal area (black arrows). (**c**) Extensive high signal area (red arrows). Scale bar = 1 mm.

Red-yellow area on OCT image represents high backscattering signal and was used to assess endometrial vascularization. Three random OCT images were selected from uterine fundus, uterine body and isthmus region, respectively, for each woman and all images were mixed before assessment. The image was divided into four quadrants (A, B, C, D) and the density of high signal area was scored with (i) score 0 if no high signal identified, (ii) score 1 if focal high signal area (<50% in a quadrant) observed (iii) score 2 if extensive high signal area (>50% in a quadrant) observed (Figure 1). Each quadrant was scored and a median score was obtained for each region, i.e., the uterine fundus, uterine body, and isthmus region.

### 2.5. Endometrial Biopsy

All participants in this study performed daily urine dipstick test from day 9 of the menstrual cycle onwards to detect the luteinizing hormone (LH) surge and endometrial biopsies were precisely obtained on day LH+7 in the non-conception cycles. A Pipet Curet (Cooper Surgical Inc., Trumbull, CT, USA) was used to obtain endometrial specimens, which were then immediately put into 10% neutral buffered formalin for over-night fixation. 

### 2.6. Hematoxylin-Eosin (H and E) Staining and Immunohistochemistry Staining 

After embedded into paraffin wax, endometrial specimens were firstly examined with routine H&E staining. For the immunohistochemistry staining, as stated in our earlier study [12], endometrial tissue sections were dewaxed in xylene and dehydrated through descending ethanol. After washing in phosphate-buffered saline, they were quenched in 0.3% hydrogen peroxide in methanol. Antigen retrieval was performed and then the sections were blocked with donkey serum. Endometrial tissue sections were incubated overnight in primary antibody (rabbit polyclonal anti-human von Willebrand factor (vWF) antibody, DAKO, Carpinteria, CA, USA, A0082 at a dilution of 1:800; rabbit polyclonal anti-human VEGF-A antibody, AbCam, Hong Kong, ab51745 at a dilution of 1:100). After incubation in secondary antibody, the specific antibody binding was visualized with the use of peroxidase substrate and counterstained with hematoxylin. In the negative control, rabbit IgG was used for the substitution for primary antibody.

### 2.7. Microvessel Counting

As stated in our previous work [13], systematic random field was selected for microvessel counting. The first field was randomly selected over the L upper corner of the microscopic field and subsequent fields were acquired by moving horizontally. A total of five completed microscopic fields for each endometrial specimen had been examined. Any brown-staining endothelial cell was regarded as a single and countable vessel. The result was expressed as MVD, which was calculated as the number of microvessels per mm^2^.

### 2.8. H-Score Analysis

H-score was used to grade and calculate the intensity of VEGF-A expression, based on the equation: H-score = ∑P_i_ (*i*+1), in which *i* represented the intensity of staining (1 = weak, 2 = moderate and 3 = strong) and P_i_ represented the percentage of cells at each intensity (0–100%). H-scores were calculated in different endometrial compartments, namely luminal epithelium, glandular epithelium, and stroma, separately. 

### 2.9. Frozen-Thawed Embryo Transfer in the Subsequent Cycle

In natural cycle, daily hormonal monitoring of estrodial/LH/progesterone was performed four days prior to the expected day of ovulation or if estrodial ≥400 pmol/L until evidence of LH surge. Ultrasound was performed in arranged in the peri-ovulatory period to measure endometrial thickness and follicle diameter. Ovulation was assumed to occur 36 hours after LH surge. Day-three embryos and blastocycsts were thawed and transferred three days and five days following the estimated day of ovulation, respectively.

In hormonal replacement treatment cycle, 6 mg oestradiol was given daily orally from day 2 of the menstrual cycle for 14 days. Transvaginal ultrasound was performed on day 14 of the estrogen therapy to assess endometrial thickness and to exclude ovarian activity. If endometrial thickness was ≥8 mm, plasma progesterone level was <4 nmol/L, progesterone was commenced using either Endometrin (Ferring, Saint-Prex, Switzerland) 100 mg TDS vaginally. Day-three embryos and blastocycsts were thawed and transferred three days and five days following progesterone administration, respectively.

### 2.10. Statistical Analysis

A Shapiro–Wilk test was used to check the data distribution. The mean ± SD was used to present the normally distributed data while median (range) was used for skewed data. The Student’s *t* test and Mann–Whitney U test were applied to compare normally distributed data and nonparametric data, respectively. The difference of qualitative data was compared by chi-square test. *p* < 0.05 was considered significant. All of the statistical analysis was performed using SPSS software (IBM, New York, NY, USA).

## 3. Results

### 3.1. Demographics

In this study, 25 women conceived, and 22 women did not conceive in the subsequent IVF-ET cycle, out of which 42 women had single embryo transferred and the other five women had double embryos transferred; in those with two embryos transferred, one woman conceived twins and four women did not conceive. A total of 38 women were transferred day-five blastocysts (21 in the pregnant group and 17 in the non-pregnant group) and nine women were transferred day-three embryos (four in the pregnant group and five in the non-pregnant group). None of the participants underwent pre-implantation genetic test (PGT) treatment. The demographic details and embryo information of these participants were summarized in Table 1. All participants had normal BMI and regular menstrual cycle. Twenty-five of them had a successful clinical pregnancy after subsequent IVF treatment, while the other 22 women were non-pregnant. There was no significant difference in mean age, FSH on Day 2, LH on Day 2 and Day 21 progesterone level between the two groups.

### 3.2. Microvessel in the Endometrium 

**Figure 2:**
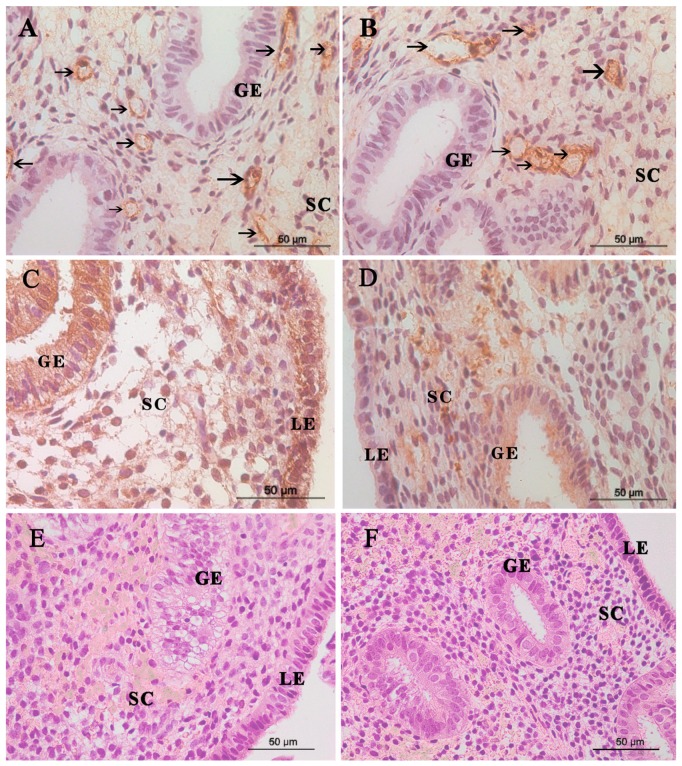
Microvessels, VEGF-A expression, and H and E staining in the endometrium. The micro blood vessels in endometrial biopsies was determined by anti-vWF–stained sections. Representative images of blood vessels localized in endometrium (arrows) from (**A**) a woman with subsequent successful pregnancy and (**B**) a woman without subsequent pregnancy are shown. VEGF-A expression in the endometrium from (**C**) a woman with subsequent successful pregnancy and (**D**) a woman without subsequent pregnancy. Representative images of H&E staining from (**E**) a woman with subsequent successful pregnancy and (**F**) a woman without subsequent pregnancy. Scale bar= 50 μm. GE, glandular epithelium; LE, luminal epithelium; SC, stromal cell. VEGF-A, vascular endothelial growth factor-A.

The immunostaining of endometrial vWF expression from pregnant women and non-pregnant women was shown in Figure 2. Positive staining for the vWF representing for endothelial cell was clearly seen in endometrial stroma, which was regarded as a single and countable vessel. The median MVD in women with subsequent pregnancy (10, range 4–17) was found to be significantly higher than that of non-pregnant women (7, range 4–10) (Table 1).

### 3.3. VEGF-A Staining

Figure 2 also showed immunostaining of VEGF-A in the peri-implantation endometrium. Positive staining for the VEGF-A was seen in all compartments of endometrium. The intensity for VEGF-A staining in stroma from women with successful subsequent pregnancy was significantly higher than that from women with failed pregnancy. There was no significant difference in H-score of VEGF-A in luminal epithelium and glandular epithelium between both groups (Table 1). 

### 3.4. H and E Staining

Representative images of H and E staining are shown in Figure 2. All endometrial tissues were consistent with secretory endometrium. No malignancy was seen.

### 3.5. OCT for the Endometrial Vascularization

**Figure 3:**
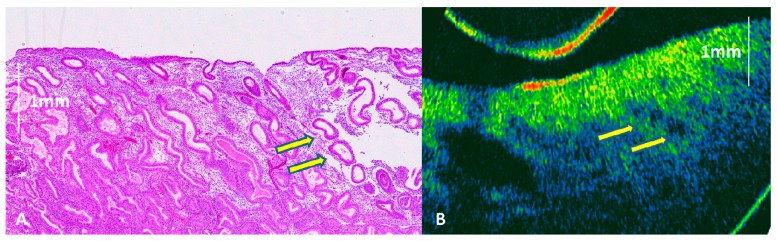
Mid-secretory phase endometrium demonstrated by parallel histology (**a**) and OCT images (**b**). Endometrial glands (yellow arrows) could be seen on OCT images.

An image of endometrium demonstrated by parallel histology and OCT image was shown in Figure 3. Based on the scoring of high signal area detected by OCT (Figure 1), the proportion of extensive high signal area from uterine body in women with successful subsequent pregnancy was significantly higher than those without pregnancy. However, there was no significant difference in the proportion of extensive high signal area from fundus and isthmus regions between both groups (Table 2).

### 3.6. Correlation between OCT Parameter and Histological Angiogenic Markers

Among the 20 women undergoing OCT imaging, a significantly higher stromal expression of VEGF-A and MVD was found in women with extensive high signal area in uterine body, compared to those with focal or no high signal area. There was no significant difference in MVD, luminal epithelial expression of VEGF-A, and glandular epithelial expression of VEGF-A between women with extensive high signal area and those with focal or no high signal area in the uterine fundus and isthmus regions (Table 3).

MVD, micro vessel density, calculated as number of micro blood vessels/mm^2^; VEGF-A, vascular endothelial growth factor-A; OCT, optical coherence tomography.

## 4. Discussion

In this study, we have used a new method, namely OCT, to evaluate endometrial vascularization and subsequently observed a significantly increased proportion of an extensive high signal area in women who were pregnant. Using immunohistochemistry, we have also found an increased MVD and higher stromal expression of VEGF-A in the peri-implantation endometrium in women with successful subsequent pregnancy during IVF-ET treatment. In addition, there was an association between the two methods of measuring endometrial vascularity.

Embryo implantation remains one of the most critical steps in determining the success of IVF treatment despite the significant advances in this field in recent years. A prerequisite for successful implantation requires vascular adaptation in the endometrium and endometrial angiogenesis has been therefore thought to be a marker of the receptive endometrium [2]. An earlier study investigated the intrauterine concentration of VEGF-A during the menstrual cycle and found the concentration increase significantly from the period of embryo implantation [14].

Using non-invasive ultrasonography combined with color Doppler, women with detectable endometrial blood flow were found to have better clinical outcomes, suggesting an adequate blood flow to endometrium is essential for successful pregnancy [15]. Moreover, endometrial and subendometrial vascularity was found to be higher in pregnant patients with live birth following ART than in those who suffered a miscarriage [16]. However, our previous study [13] showed that endometrial vascularity assessed by 3D ultrasound combined with power Doppler could not accurately reflect changes in micro vessel in the endometrial and sub-endometrial regions. In addition, it is still uncertain to what extent aberrant vascular adaptation is a cause of implantation failure. 

OCT combined with angiography has been widely used in ophthalmology to provide retinal and choroidal vasculature and structure [17,18]. However, there is not any previous study using OCT to determine endometrial vasculature. To the best of our knowledge, the present work is the first one to simultaneously employ both OCT and immunohistochemistry and to measure endometrial vascularization in women undergoing IVF-ET treatment. In this study, it is of interest that differences in pregnancy rates in OCT measurements are only significant when extensive high signal area takes place in the uterine body rather than uterine fundus or isthmic region. The exact underlying reason for this observation is unclear. Unlike the real-time OCT imaging, Pipelle sampling for endometrial biopsy in this study did not allow us to separate the specimen from different regions of the uterus, although most of the specimens are thought to be coming from the body of the uterus. To overcome this limitation, further study could be carried out using hysteroscopy with direct endometrial sampling over different sites of endometrium and correlate with OCT images. It would also allow measurement of MVD and VEGF-A of endometrium from different regions of uterus. With robust advance in OCT development, imaging probe with higher resolution may be able to detect micro vessels on endometrium and further study could be performed to correlate OCT images with histology.

One previous study has examined the predictive value of endometrial micro vessel for IVF treatment outcome [9]. The authors found that endometrial MVD in clinical pregnancy group was significantly higher than it in non-pregnant group. They also used a receiver operating characteristic (ROC) for analysis and the result showed a low specificity of endometrial MVD to predict clinical outcome [9]. In our current study, we have also found that women who achieved a clinical pregnancy after subsequent IVF treatment had an increased angiogenic profile in the peri-implantation endometrium, by both immunohistochemical staining and OCT methods. It seemed likely that women with failed pregnancy subsequently appear to have less endometrial angiogenesis and an inadequate of blood supply around the time of embryo implantation.

Embryo implantation is a dynamic process, which consists of apposition, adhesion, and invasion [19]. In the stromal compartment of the endometrium, where the embryo gets in close contact with maternal circulation, we have found that MVD and stromal expression of VEGF-A was significantly higher in women with successful subsequent pregnancy. However, the expression of VEGF-A in luminal epithelium and glandular epithelium did not differ between both groups. Taken these observations together, we can infer that an inadequate angiogenesis in the stromal compartment was associated with impairment of implantation.

Whilst we have demonstrated altered endometrial vascularization in women undergoing IVF-ET treatment, there are still several unanswered questions yet. Firstly, given that it has been considered that early embryo development requires a relatively low oxygen environment [20], it is unclear how the decreased MVD could be detrimental to the impending embryo implantation. We have demonstrated association but it is not clear if the association is casual or causal. Secondly, all the women studied were in non-conception cycles. It is not certain whether the same observation would persist in the peri-implantation period of another menstrual cycle or in a conception cycle. Thirdly, since the possible causes of infertility could be various, the selected population would be less homogenous. Therefore, further studies are required to confirm our finding especially in relation to OCT which is non-invasive method of investigation and which can be repeated serially.

There are several limitations of this study. One possible criticism of our study was that observations in the non-conception endometrial biopsy cycle (four weeks prior to embryo replacement) may not be exactly the same as the endometrium at the time of embryo transfer, given that there some degree of cycle to cycle variation may occur [21]. Moreover, it is also possible that there are subtle differences in progesterone profile and endometrial receptivity between these two types of cycle [22] and the inclusion of two types of embryo transfer cycle, namely natural and artificial (hormone replacement) cycles, could have introduced an additional confounding variable. The possible impact of the progesterone level on the day of hCG trigger could well be another confounding variable [23], but it is unlikely to be present given that embryos were not replaced in ovarian stimulation cycles. Secondly, vWF was used as an endothelial marker to identify microvessels in the endometrium in this study. However, the examination of vascularization could be improved by the use of double immunostaining for the marker of endothelial cells (CD34 or vWF) and marker of perivascular smooth muscle (smooth muscle α actin). In addition, while we have recruited 47 women in this study, only 20 of them underwent OCT. We acknowledge that the sample size was relatively small but we considered it adequate for a pilot study. In any case, the findings that were significant in OCT measurements between the two groups, even with a small sample size, suggested that the difference was not a subtle one. However, given that it is the first report of this finding, subsequent studies with larger sample size are necessary.

## 5. Conclusions

To conclude, both immunohistochemistry and OCT demonstrated significant difference in vascularization of the peri-implantation endometrium between subjects who did and did not conceive after IVF-ET treatment. Our findings also suggest OCT appears to be a promising non-invasive or minimally invasive alternative to study endometrial vascularity in women with reproductive failure.

## Figures and Tables

**Table 1:** Demographics data and histologically endometrial angiogenic markers on day LH+7 in women undergoing IVF-ET treatment (*n* = 47).

Parameter	Pregnant (*n* = 25)	Non-Pregnant (*n* = 22)	*p* Value
Age, mean ± SD, years	33.9 ± 3.2	34.7 ± 3.5	0.072
FSH on Day 2, mean ± SD, IU/L	6.2 ± 1.1	7.2 ± 1.6	0.069
LH on Day 2, mean ± SD, IU/L	5.4 ± 1.0	5.8 ± 1.0	0.151
Progesterone on Day 21, mean ± SD, nmol/l	47.8 ± 11.2	45.9 ± 13.3	0.089
Morphological grading of embryo transferred on day 3			
Grade 1, *n/n* (%)	2/4 (50)	2/5 (40)	1.000
Grade 2, *n/n* (%)	2/4 (50)	3/5 (50)	
Morphological grading of embryo transferred on day 5			
3BB or above, *n/n* (%)	16/21 (76)	12/17 (71)	0.494
Below 3BB, *n/n* (%)	5/21 (24)	5/17 (29)	
Endometrial preparation for frozen embryo transfer cycle			
Natural cycle, *n/n* (%)	12/25 (48)	11/22 (50)	1.000
Hormonal replacement cycle, *n/n* (%)	13/25 (52)	11/22 (50)	
MVD, median (range), number of micro vessels/mm^2^	10 (4–17)	7 (4–10)	0.021
Expression of VEGF-A in luminal epithelium, median (range), H-score	219 (62–305)	200 (106–288)	0.056
Expression of VEGF-A in glandular epithelium, median (range), H-score	193 (50–296)	157 (22–297)	0.082
Expression of VEGF-A in stroma, median (range), H-score	189 (72–395)	125 (86–299)	0.037

Normally distributed continuous data were compared by Student *t* test. Non-normally distributed data were compared by Mann-Whitney U test. Categorical data were compared by chi-square test. IVF-ET, in vitro fertilization-embryo transfer; FSH, follicular stimulating hormone; LH, luteinizing hormone; MVD, micro vessel density, calculated as number of micro blood vessels/mm^2.^; VEGF-A, vascular endothelial growth factor-A.

**Table 2:** Proportion of high signal area determined by optical coherence tomography (OCT) (*n* = 20).

Parameter	Pregnant (*n* = 10)	Non-Pregnant (*n* = 10)	*p* Value
**Uterine fundus**			
Extensive high signal area, *n* (%)	4 (40)	5 (50)	0.500
Focal or no high signal area, *n* (%)	6 (60)	5 (50)	
**Uterine body**			
Extensive high signal area, *n* (%)	8 (80)	3 (30)	0.035
Focal or no high signal area, *n* (%)	2 (20)	7 (70)	
**Isthmus region**			
Extensive high signal area, *n* (%)	7 (70)	10 (100)	0.105
Focal or no high signal area, *n* (%)	3 (30)	0 (0)	

Difference between groups was compared by chi-square test.

**Table 3:** Correlation between OCT parameters and endometrial histological angiogenic markers in women undergoing IVF-ET treatment (*n* = 20).

Parameter	MVD (Number of Micro Vessels/mm^2^)	VEGF-A in Luminal Epithelium, (H-Score)	VEGF-A in Glandular Epithelium, (H-Score)	VEGF-A in Stroma, (H-Score)
**Uterine fundus**				
Extensive high signal area	8 (6–12)	162 (62–305)	147 (48–280)	189 (90–333)
Focal or no high signal area	8 (4–16)	158 (86–286)	139 (55–236)	162 (84–395)
*p* value	0.106	0.340	0.223	0.068
**Uterine body**				
Extensive high signal area	9 (5–16)	185 (88–295)	155 (50–256)	196 (84–395)
Focal or no high signal area	6 (4–11)	178 (62–305)	152 (48–280)	135 (92–302)
*p* value	0.041	0.197	0.068	0.039
**Isthmus region**				
Extensive high signal area	7 (4–15)	177 (62–278)	144 (60–280)	176 (94–395)
Focal or no high signal area	7 (5–16)	172 (76–305)	159 (48–276)	177 (84–320)
*p* value	0.452	0.129	0.098	0.076

Data was shown as median (range). Difference between groups was compared by non-parametric Mann-Whitney U test.
